# Pedigree derived mutation rate across the entire mitochondrial genome of the Norfolk Island population

**DOI:** 10.1038/s41598-022-10530-3

**Published:** 2022-04-26

**Authors:** J. R. Connell, M. C. Benton, R. A. Lea, H. G. Sutherland, J. Chaseling, L. M. Haupt, K. M. Wright, L. R. Griffiths

**Affiliations:** 1 Centre for Genomics and Personalised Health, Genomics Research Centre, School of Biomedical Sciences, Queensland University of Technology (QUT), 60 Musk Ave., Kelvin Grove, QLD 4059 Australia; 2 School of Environment and Science, Griffith University, Nathan, QLD Australia; 3 Unrecovered War Casualties-Army, Australian Defence Force, Russell Offices, Russell, Australian Capital Territory Australia; 4 Royal Australian Air Force (RAAF), No 2 Expeditionary Health Squadron, Williamtown, NSW Australia; 5 Present Address: Human Genomics, Kenepuru Science Centre, Institute of Environmental Science and Research, Wellington, New Zealand

**Keywords:** Evolutionary genetics, Phylogenetics, Population genetics, Genetic markers, Genomics, Mutation, Sequencing

## Abstract

Estimates of mutation rates for various regions of the human mitochondrial genome (mtGenome) vary widely, depending on whether they are inferred using a phylogenetic approach or obtained directly from pedigrees. Traditionally, only the control region, or small portions of the coding region have been targeted for analysis due to the cost and effort required to produce whole mtGenome Sanger profiles. Here, we report one of the first pedigree derived mutation rates for the entire human mtGenome. The entire mtGenome from 225 individuals originating from Norfolk Island was analysed to estimate the pedigree derived mutation rate and compared against published mutation rates. These individuals were from 45 maternal lineages spanning 345 generational events. Mutation rates for various portions of the mtGenome were calculated. Nine mutations (including two transitions and seven cases of heteroplasmy) were observed, resulting in a rate of 0.058 mutations/site/million years (95% CI 0.031–0.108). These mutation rates are approximately 16 times higher than estimates derived from phylogenetic analysis with heteroplasmy detected in 13 samples (n = 225, 5.8% individuals). Providing one of the first pedigree derived estimates for the entire mtGenome, this study provides a better understanding of human mtGenome evolution and has relevance to many research fields, including medicine, anthropology and forensics.

## Introduction

The mitochondrial genome (mtGenome), and the control region in particular, is the most analysed DNA sequence in human evolutionary studies. As a result, the rate of change in mitochondrial DNA (mtDNA) is relatively well understood. However, since the 1990s, evidence has emerged that mtDNA mutation rates vary widely and are dependent upon the estimation method used and the population studied (e.g. when comparing Swedes and Icelanders, respectively, in Cavelier et al.^1^ and Siguroardottir et al.^2^.

The rate at which mtDNA mutates is predominantly estimated using phylogenetic or pedigree methods. Phylogenetic analysis of mtDNA relies upon haplotype trees and phylogenetically derived divergence rates, while human pedigree-based mutation rates are estimated by comparison of parent/offspring pairs or using deep-rooted familial lineages at particular loci and counting the number of novel mutations per pair, and this value is then divided by the number of meioses^3^. Discrepancy exists between the estimates produced using these two methods, although recent progress in the analysis of ancient DNA has allowed for further improvement in the estimates^4–6^. Since the first noted^7^, the cause of this discrepancy has been extensively explored and several plausible causes have been suggested, namely: differences in the mutation rate at different locations within the mtGenome; sample size and selection; the effect of natural selection and genetic drift; the occurrence of somatic mutations; the unintended sequencing of nuclear mitochondrial pseudogenes (NUMTs); and the leakage of paternal mtDNA and recombination^2,8–13^. Thus, the choice of which estimate to use in population studies is determined by the specific purpose of the investigation. Phylogenetically based estimations may be more suited to investigations of deep history as this considers mutations that have reached a considerable frequency in the population. In contrast, pedigree-based estimations may be more suited to studies of recent history as newly acquired mutations that have not had time to become fixed within the population are considered^12,14^.

The human mtGenome is split into two sections: a large coding region that is responsible cellular energy production and for the gene production for transfer RNA (tRNA), ribosomal RNA (rRNA) translation; and a smaller control region^15^. The control region also contains the polymorphism-rich hypervariable regions (HVI and HVII), which are traditionally used for comparisons in missing persons cases, criminal cases and for historical military cases^16^. While the mitochondrial control region has been extensively studied, minimal literature explores the mutation rate for the entire mtGenome. Typically only the control region (or portions of) are examined due to the cost and effort required to produce whole mtGenome Sanger profiles and the perceived stability of the remainder of the genome. The advent of Next Generation Sequencing (NGS) technologies has provided impetus and the potential to expand the current body of knowledge surrounding the prediction of mutation rates.

In an effort to estimate the mutation rate of the entire human mtGenome using a non-phylogenetic approach, we utilised 225 members of the most recent four generations of the Norfolk Island (NI) core pedigree^17–21^ and compared entire mtGenome sequences of maternal relatives from 45 maternal lineages. NI is a small, remote island in the Pacific with a unique admixture of paternal European ancestry in combination with Polynesian maternal origins^22^. The population has a well-documented history and genealogy. Accurate and detailed historical accounts have been used by genealogists to create and maintain a well-documented, large multigenerational NI pedigree which consists of 5742 individuals spanning 11 generations, and 200 years to the original founders^23^. Due to the many inbreeding loops found in the population’s early generations, and the size and complexity, the NI pedigree was reconstructed to include all core individuals relating back to the original founders (n = 1388)^21^.

This study defines one of the first pedigree derived mutation rates encompassing the entire human mtGenome. As with Sigurðardóttir et al.^2^, we define mutation rate as the rate at which the mtDNA of an individual changes, rather than the rate of sequence change at the level of an individual mitochondria. The expansion of the current paradigms and evidence of mutation rates allows researchers to more fully understand the processes that have shaped the evolution of the human mtGenome. Our findings have application and implications for various research fields including clinical genetics, human evolution and forensic identity testing.

## Results

### Whole mtGenome sequencing to identify mtDNA variants in the Norfolk Island pedigree

**Figure 1:**
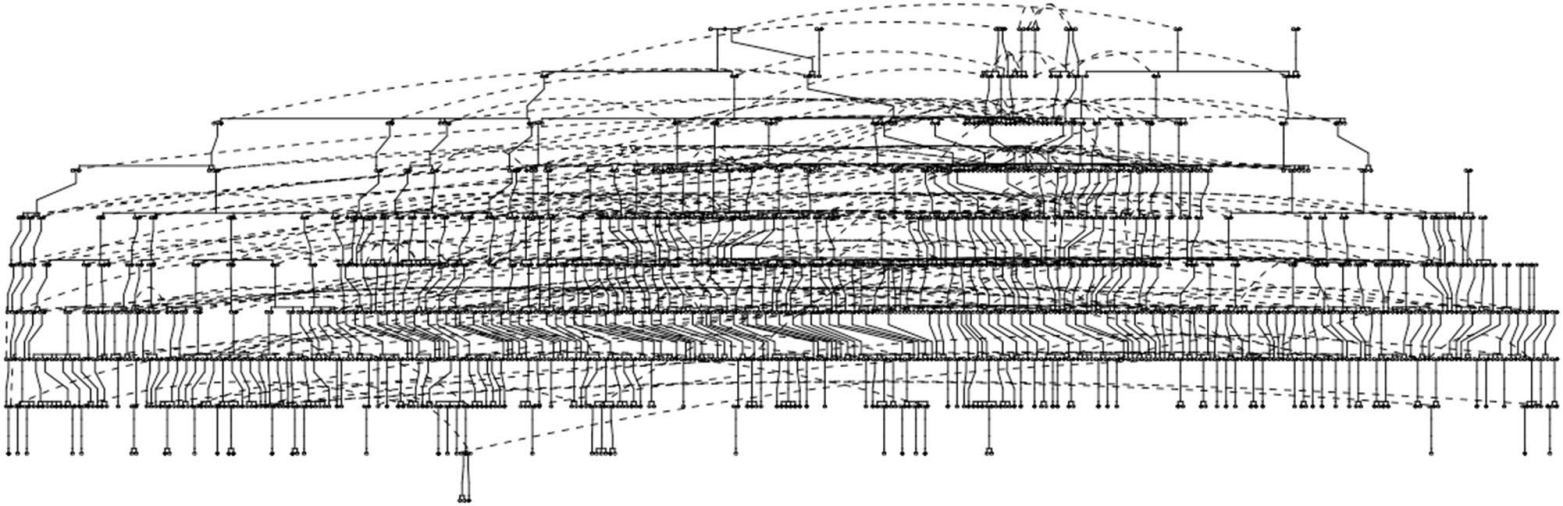
Norfolk Island core pedigree. Reconstruction of the original Norfolk Island pedigree, based on available genealogical and genetic information (*n* = 1388). The core pedigree spans 11 generations and contains individuals who directly relate back to the original founders of Norfolk Island. Figure adapted from^21^ and generated using the Pedigree v1.4^54^, Kinship2 v1.8.5^55^ and Tidyverse v1.3.1^56^ packages in RStudio v1.4^57^.

The whole mtGenome was sequenced for 225 individuals representing 45 independent mtDNA lineages from the core pedigree of the NI population isolate on an Ion Torrent NGS platform (Fig. 1). Sequence quality (Phred) scores remained consistent at > 25 for all samples at the median read length (140 bp), with data reaching a median depth of approximately 370X for all 225 samples. Sequencing reads were aligned in relation to the rCRS and variants annotated using Mitomaster, with called variants subsequently validated by Sanger Sequencing.

In this study, NGS analysis of homopolymer stretches (such as poly C regions) were a challenge for the Ion Torrent platform resulting in sequencing slippage in regions containing long homopolymer stretches. This is due to the degree of change in voltage loss resolution (above 6–8 bp) inherent to the platform. As a result, these regions have poor mapping quality and can lead to false-positive and false-negative calls and typically require assessment using alternative methods. The identification and calling INDELS also presented a bioinformatic challenge in the samples due to sequencing error bias and required a higher level of manual assessment. Issues in sequencing data in homopolymeric regions are not unique to NGS. Sanger sequencing is also susceptible to such errors^24^, with all sequencing platforms prone to sequencing errors corresponding to the chemistry and platforms used^25^.

In the rCRS, position T16189 is flanked by cytosine residues between positions 16183 and 16194. In the samples examined in this study, this sequence stretch was consistently and reproducibly represented in NGS and Sanger data analysis when no length or point variation was present. However, in 134 samples (n = 225, 59.6%), a combination of the T16189C transition and a deletion at position 16189 resulted in low coverage values and unreliable Sanger data for this region. Consequently, it became difficult to determine the number of cytosine residues present. In accordance with Interpretation Guidelines for mtDNA Sequencing outlined by the Laboratory Division of the FBI^26^, no attempt was made to count the number of residues for interpretation purposes and all comparisons assumed the same number were present. Similar results were obtained for the HVII C-track between position 302 to 310 and 310 to 316. Difficulties were also observed when an insertion of one or more Cs (resulting in a stretch of ≥ 8 C residues) were present. Mutations in these regions were omitted during the comparison of maternally related individuals, and hence were not included in the mutation rate calculations performed in this study.

### Calculation of mutation rates across the mtGenome

When the individual pedigrees were examined, the number of meioses and mutations were observed (Table 1). Homoplasmic mutations were found at two different sites in one individual each. Heteroplasmy was confirmed at 7 positions across 13 individuals (n = 225, 5.78% individuals). Analysis of the individuals belonging to 45 families from NI revealed 13.3% of the families contained at least one individual with mtDNA heteroplasmy as a result of mutation across the entire mtGenome. The frequency of point heteroplasmy in the NI samples did not show significant differences in relation to gender (Chi-squared test: X^2^ = 0.472, *df* = 1, P = 0.492), nor was it found to be associated with a specific mtDNA haplogroup (Fisher exact test: P = 0.080).

**Table 1:** Mutations observed in 45 maternal lineages.

Pedigree ID	Number of meioses in the pedigree	Number of individuals analysed	Number of mutations observed	Number of individuals with mutation	Mutations
13	14	10	2	1, 3	A8470R, A16280R
23	10	7	0	–	–
167	3	3	0	–	–
208	7	7	1	3	T9012Y
219	4	3	0	–	–
242	13	5	0	–	–
490	8	4	1	1	T146Y
691	130	59	2	2, 1	A2833R, A8817G
694	63	29	0	–	–
833	2	2	0	–	–
1088	3	4	0	–	–
1597	15	7	1	1	A16247G
1878	10	7	0	–	–
2071	5	4	0	–	–
2212	2	2	0	–	–
2328	2	2	0	–	–
2462	4	2	0	–	–
2802	3	3	0	–	–
2913	2	2	0	–	–
3310	4	3	0	–	–
3363	2	2	0	–	–
3963	3	3	0	–	–
5581	2	2	0	–	–
110390	1	2	0	–	–
124570	1	2	1	1	C16344Y
125750	2	3	0	–	–
169040	1	2	0	–	–
169810	1	2	0	–	–
218290	3	2	0	–	–
301005	1	2	0	–	–
310881	1	2	0	–	–
311021	2	3	0	–	–
313181	1	2	0	–	–
313211	1	2	0	–	–
313851	1	2	0	–	–
315560	1	2	0	–	–
317611	1	2	0	–	–
317671	2	3	0	–	–
317741	1	2	0	–	–
319011	1	2	0	–	–
319271	2	3	0	–	–
320321	2	3	0	–	–
321870	2	3	0	–	–
329180	4	5	1	2	C16320Y
400099	2	2	0	–	–
Total	345	225	9		

Heteroplasmy are designated with an International Union of Biochemistry code^27^.

Further analysis of the 345 mtDNA transmissions identified 9 mutations (2 transitions and 7 cases of heteroplasmy) across the entire mtGenome, suggesting that 1.57 × 10^–6^ mutations occur (at a detectable level) in the mtGenome in each generation. Assuming a generation time of 26.9 years, the mutation rate for the entire mtGenome would be 0.058 mutations/site/Myr when including heteroplasmy (95% CI 0.031–0.108), and 0.013 mutations/site/Myr when excluding heteroplasmy (95% CI 0.003–0.047) (Table 2). In order to make an estimate of separate mutation rates for the HVI and HVII regions, HVI was delimited by positions 16024–16383 (360 bp) and HVII delimited by positions 57–371 (315 bp) in accordance with Sigurgardottir et al.^2^.

**Table 2:** Mutation rates for the NI extended pedigree.

	Point estimate	95% CI Clopper Pearson		95% CI Wilson	
		Lower	Upper	Lower	Upper
mtGenome (1–16596)	0.058	0.026	0.110	0.031	0.108
HVI (16024–16383)	1.201	0.338	3.039	0.473	3.039
HVII (57–371)	0.343	0.000	1.930	0.077	1.930
HVI/HVII (16024–16383 and 57–371)	0.801	0.252	1.837	0.360	1.837
Control region (16024–576)	0.469	0.148	1.077	0.211	1.077
Coding region (577–16023)	0.028	0.008	0.071	0.011	0.071

Pedigree derived mutation rate calculated for various regions of the mtGenome. Rates are expressed in mutations per site per million years (26.9 year generation time).

mtGenome, mitochondrial genome; HVI, hypervariable region 1; HVII, hypervariable region II. All the mutations (including heteroplasmy) that were detected were considered.

## Discussion

### Mutation rate

In the analysis of 345 genetic transmissions, 9 mutations (2 transitions and 7 cases of heteroplasmy) were detected across the entire mtGenome, suggesting the mutation rate for the entire mtGenome to be 0.058 mutations/site/Myr (95% CI 0.031–0.108). Table 3 compares the derived mutation rate for each region calculated using the NI samples with previous studies using closed populations. NI sample derived mutation rates are shown in bold. For consistency, mutation rates for each study were converted to mutations/site/Myr with a defined generation time of 25 years. Only rates that could be accurately standardised were included in the table. As such, a number of published rates were excluded, for example those by^28–35^.

**Table 3:** Summary of the derived mtDNA mutation rate for various published studies.

Region	Study	Type	Sequence range	mtDNA transmissions	Sub^a^	Hetero^b^	Total^c^	Reported mutation rate	Standardised mutation rate*
HVI	^9^	Pedigree	16024–16383	321				1.84 × 10^–6^ mut/site/generation^d^	0.074^d^
	^38^	Phylogenetic	16024–16401					10.3 × 10^–8^ mut/site/year^m^	0.103
	^1^	Pedigree	Amplification range: L15997 and H202	292	0			0.45 mut/site/Myr^f,g^	0.360^f^
	^2^	Pedigree	16024–16383	705	3	3	6	–	0.948^e^
	**Current**	**Pedigree**	**16024**–**16383**	**345**	**1**	**3**	**4**	**1.201 mut/site/Myr**^**e**^	**1.292**^**e**^
	^9^	Pedigree	16024–16383	321	0	6	6	–	2.083^e^
	^36^	Pedigree	16024–16383	299			7	71.1 × 10^–6^ mut/site/generation^h^	2.844^ h^
HVII	^38^	Phylogenetic	29–408					7.39 × 10^–8^ mut/site/year^m^	0.074
	^2^	Pedigree	57–371	705	1	0	1	–	0.181^f^
	^1^	Pedigree	Amplification range: L16483 and H580	291	0	0	0	0.42 mut/site/Myr^fg^	0.336f.
	**Current**	**Pedigree**	**57**–**371**	**345**	**0**	**1**	**1**	**0.343 mut/site/Myr**^**e**^	**0.369**^**e**^
	^9^	Pedigree	57–371	321				10.83 × 10^–6^ mut/site/generation^d^	0.433^d^
	^9^	Pedigree	57–371	321	0	5	5	–	1.984^e^
HVI/HVII	^1^	Pedigree	Amplification range: L16483 and H580	291	0	0	0	0.21mut/site/Myr^fg^	0.168^f^
	^9^	Pedigree	57–371 and 16024–16383	321				6.04 × 10^–6^ mut/site/generation^d^	0.241^d^
	^2^	Pedigree	57–371 and 16024–16383	705				0.32 mut/site/Myr^f,g^	0.256^f^
	^39^	aDNA	Undefined					31.43 × 10^–8^ μ/site/year	0.314
	^2^	Pedigree	57–371 and 16024–16383	705	3	3	6	–	0.506^e^
	**Current**	**Pedigree**	**57–371 and 16024–16383**	**345**	**1**	**4**	**5**	**0.801 mut/site/Myr**^**e**^	**0.861**^**e**^
	^11^	Pedigree	Undefined	327			10	2.5/site/Myr^e,g^	2.000^e^
	^9^	Pedigree	57–371 and 16024–16383	321	0	11	11	–	2.037^e^
Control region	^40^	Pedigree	Undefined					1.5 × 10^–6^ mut/site/generation	0.060
	^38^	Phylogenetic	Undefined					7.00 × 10^–8^ mut/site/year^m^	0.070
	^9^	Pedigree	1–400 and 16024–16569	321				4.19 × 10^–6^ mut/site/generation^d^	0.168^d^
	^1^	Pedigree	Amplification range: L15997 and H202, with L16483 and H580	292	0	0	0	0.21 mut/site/Myr^f,g^	0.168^f^
	^10^	Pedigree	1–576 and 16024–16596	185			1	0.24 mut/site/Myr^g,i^	0.188^i^
	**Current**	**Pedigree**	**1–576 and 16024–16569**	**345**	**1**	**4**	**5**	**0.469 mut/site/Myr**^**e**^	**0.505**^**e**^
	^9^	Pedigree	1–400 and 16024–16569	321				1.28 × 10^–5^ mut/site/generation^j^	0.512^j^
	^9^	Pedigree	1–400 and 16024–16596	321	0	6	6	1.92 × 10^–5^ mut/site/generation^i^	0.768^i^
	^9^	Pedigree	1–400 and 16024–16569	321	0	11	11	3.52 × 10^–5^ mut/site/generation^e^	1.194^e^
Coding Region	^41^	aDNA	577–16023					1.25 ± 0.68 × 10^–8^ sub/site/year^o^	0.0125 ± 0.0068
	^42^	Phylogenetic	Undefined					1.70 × 10^–8^ sub/site/year^n^	0.017
	^43^	Pedigree	3230–4331	311				5.89 × 10^–7^ mut/site/generation^d^	0.024^d^
	**Current**	**Pedigree**	**576**–**16024**	**345**	**1**	**3**	**4**	**0.028 mut/site/Myr**^**e**^	**0.030**^**e**^
	^43^	Pedigree	3230–4331	311				1.03 × 10^–6^ mut/site/generation^l^	0.041^ l^
	^43^	Pedigree	3230–4331	311				5.84 × 10^–6^ mut/site/generation^i^	0.234^i^
	^43^	Pedigree	3230–4331	311				5.84 × 10^–6^ mut/site/generation^j^	0.234^j^
	^43^	Pedigree	3230–4331	311	0	3	3	8.75 × 10^–6^ mut/site/generation^e^	0.350^e^
	^43^	Pedigree	3230–4331	311				8.75 × 10^–6^ mut/site/generation^k^	0.350^ k^
	^1^	Pedigree	5550–6550	256	0	0	0	0.54 mut/site/Myr^f,g^	0.432
Complete mtGenome	^40^	Pedigree	1–16569					2.7 × 10^–7^ mut/site/generation^o^	0.011
	^41^	aDNA	Undefined					1.92 × 10^–8^ sub/site/year^o^	0.019
	^39^	aDNA	Undefined					2.143 × 10^–8^ μ/site/year	0.021
	^44^	aDNA	Undefined					2.4 × 10^−8^ substitutions/site/year	0.024
	**Current**	**Pedigree**	**1–16569**	**345**	**2**	**7**	**9**	**0.058** **mut/site/Myr**^**e**^	**0.063**^**e**^

*mut/site/Myr—mutations per nucleotide per million years. Mutation rates have been adjusted for consistent comparison. One generation is 25 years.

Sub, substitutions; Hetero, heteroplasmic mutations; aDNA, ancient DNA.

^a^Number of homoplasmic mutations observed.

^b^Number of heteroplasmic mutations observed.

^c^Total number of homoplasmic and heteroplasmic mutations observed.

^d^Only the substitutions (including heteroplasmy) with a germinal origin present in women that would become fixed at the individual level were considered.

^e^All the substitutions (including heteroplasmy) that were detected were considered.

^f^Only homoplasmic mutations were considered.

^g^One generation is 20 years.

^h^Unclear if heteroplasmy included in rate.

^i^Only the substitutions (including heteroplasmy) for which there was evidence of a germinal origin were considered.

^j^Only the substitutions (including heteroplasmy) present in women for whom there was evidence of a germinal origin were considered.

^k^Only the substitutions (including heteroplasmy) present in women were considered.

^l^Only the substitutions (including heteroplasmy) present in women that would become fixed at the individual level considering neutrality were considered.

^m^Date of divergence human-chimpanzee used to calibrate evolutionary rate: 4.9 million years.

^n^Date of divergence human-chimpanzee used to calibrate evolutionary rate: 5 million years.

^o^Date of divergence human-chimpanzee used to calibrate evolutionary rate: 6.5 million year.

Note that although rates are grouped based on region (such as control region, coding region), the sequence range varies from study to study. Furthermore, inclusion requirements varied for each study impacting the rate calculated (for example, only substitutions for which there was evidence of a germinal origin were considered in Santos et al.^9^, or all substitutions detected were considered in Madrigal et al.^36^). As expected, the mutation rate for the coding region was found to be smaller than that reported for the control region, and as with other studies, the estimation reported here is much higher than those obtained by phylogenetic methods (Table 3).

In the NI samples, with the exception of the coding region, the mutation rate calculated for various regions of the mtGenome were found to be relatively high when compared to previous published studies. This may be attributed to differences in the population examined, study design or analysis. In this study, the rates calculated for the NI population included all mutations (heteroplasmy, germline, somatic). In contrast, for the HVI region, Santos et al.^9^ reported a mutation rate of 0.074 mutations/site/Myr (16 times lower than the rate for NI: 1.2 mutations/site/Myr–both using a generation time of 25 years), however their rate only included substitutions (including heteroplasmy) with a germinal origin present in women fixed at the individual level. While inclusion criteria alone does not fully explain the variations identified via pedigree-derived mutation rates, it likely contributes to the observed difference.

Interestingly, the mutation rate for the coding region was one of the smallest rates reported, despite our study being the only one to examine the entire region (bases 576–16024). This variation could be due to population differences. NI is a small, isolated population, and therefore the evolution of extremely high mutation rates is unlikely to occur unless organisms are under special circumstances^37^, with beneficial mutations rarely compensating for deleterious mutations.

In addition, heteroplasmic variants were not included in all studies assessing the mutation rate of mtDNA (for example^1^). It is reasonable to suggest that heteroplasmy may resolve in favour of a ‘new’ base, and therefore, exclusion of heteroplasmic variants may underestimate the true mutation rate. However, the inclusion of heteroplasmic mutations introduces the limitation that only those that have reached a level of 20% of the mtDNA population are deemed detected. This raises the question of whether the discrepancy between phylogenetic and pedigree derived rates could be answered by simply excluding heteroplasmic variants, on the presumption that they will not become homoplasmic and/or will not be transmitted at the level of the population. Heteroplasmic mutations that reached 20%, the minimum threshold required to be included in the pedigree derived mutation rate reported here, should have a higher probability of becoming homoplasmic than those reaching only 1–2%.

### Heteroplasmy

Several previous studies have demonstrated Sanger sequencing to be valid for quantification of heteroplasmy greater than 10% and that NGS could detect and quantify heteroplasmy as low as 1%^45^. In this study, to ensure robustness of the data and subsequent analysis, a threshold of 20% or greater was used. Previous studies have also performed independent DNA extraction, PCR amplification and sequencing to authenticate heteroplasmy results and exclude any contamination^9,46,47^. Here, heteroplasmy was confirmed via Sanger sequencing.

Point heteroplasmy was confirmed at 7 positions across 13 samples (n = 225, 5.8% individuals), a rate found to be lower than other studies that have previously sequenced the entire mtGenome. Ramos et al.^46^ observed point heteroplasmy in 12.8% of samples (13 of 101 individuals), while Santos et al.^9^ observed point heteroplasmy in 12.1% of samples (28 of 232 individuals). Neither of these studies used NGS and both methodologies relied upon the same method to quantify levels of heteroplasmy. Specifically, these studies measured the height of the two peaks directly from the sequencing electropherogram and calculated the proportion between the heights of each peak with respect to the sum of the height of the two peaks. For comparison purposes, the obtained numerical proportions were then averaged. Another method previously used included cloning PCR products encompassing the D-loop region and subsequent sequencing of the 26–66 clones to determine the number containing each mtDNA variant^9,46^. The increased percentage of heteroplasmy observed in those studies when compared to ours could be due to the lower minor variant threshold used. The threshold used in Ramos et al.^46^ was 10%, and although no threshold was stated in Santos et al.^9^, that study included cases of heteroplasmy where the minor variant was as low as 2.5%. In addition to those outlined in Table 1, heteroplasmic mixtures below the 20% threshold were observed in three additional individuals, with MAFs of 12, 15 and 15% for A16280R, T9012Y and C16320, respectively. As these were below the 20% threshold, they were not considered in any further analysis. Further heteroplasmic variants may be evident at other positions across the mtGenome if our threshold was reduced to one comparable with Ramos et al.^46^ or Santos et al.^9^. In our study, 6.7% (n = 45) of the NI pedigrees examined, at least one individual within the lineage presented mtDNA heteroplasmy produced by mutations across the coding region. This value is consistent with the percentage reported by Santos et al.^43^ (6.5% of families). Interestingly, no individuals from the NI pedigrees examined in this study showed heteroplasmy at the positions examined by Santos et al.^43^.

Length heteroplasmy is typically observed in every individual where a transition at position T16189 results in a homopolymer of nine or more cytosine residues, with no length heteroplasmy observed when seven or fewer cytosine residues are present^48–51^. In the NI samples, consistent and reliable Sanger sequencing was not achieved for individuals that exhibited the T16189 transition and therefore, length heteroplasmy could not be confirmed. Hence, to ensure consistent reporting, the decision was made to disregard variants in positions 16180–16183 for all samples. The poly-C tracks of both the HVI and HVII regions are known to have high insertion/deletion rates, resulting in length heteroplasmy^52^. For example, Santos et al.^9^ reported length heteroplasmy produced by the insertion of cytosine residues in the poly-C tract of HVRI and HVRII respectively in 22.92% and 54.16% of the families studied. It is accepted that the general mechanism for generating length heteroplasmy in these regions is replication slippage^53^. These regions were also excluded during the analysis of the NI individuals and no other length heteroplasmy in any region was observed in the NI samples.

## Conclusion

The mtDNA control region is commonly used to assess human evolution and population movements. Many of these studies rely on phylogenetic analysis of mtDNA control region haplotype trees and phylogenetically derived rates of divergence. Estimates of the mutation rate with a non-phylogenetic approach (namely pedigree analysis) are reported to be approximately ten-fold higher than phylogenetically derived rates. Using a pedigree approach, 9 mutations (2 transitions and 7 cases of heteroplasmy) were identified across the entire human mtGenome, resulting in a mutation rate (obtained by employing the same definition of mutation used by other authors) of 0.058 mutations/site/Myr (95% CI 0.031–0.108). The mutation rate produced from this study is one of the first to use extended pedigree analysis of the entire human mtGenome in combination with NGS. If mutations arise randomly in the mtGenome, these results suggest that newly arising mutations in the human mitochondrial coding region are eliminated before they reach a detectable frequency. Defining this mechanism may provide further insight into the interpretation of human mtGenome data across numerous fields of study.

## Materials and methods

### Ethics

Ethical clearance for the Norfolk Island mitochondrial DNA analysis portion of this study was provided originally by the Griffith University Human Research Ethics Committee (Approval MSC/04/09/HREC). Ethical clearance was transferred to and is now provided by the Queensland University of Technology Human Research Ethics Committee (Approval Number: 1400000749). No other ethical clearance was required. All analyses were performed in accordance with relevant guidelines and regulations, and all Norfolk participants provided informed consent for research involvement.

### Sample selection

**Figure 2:**
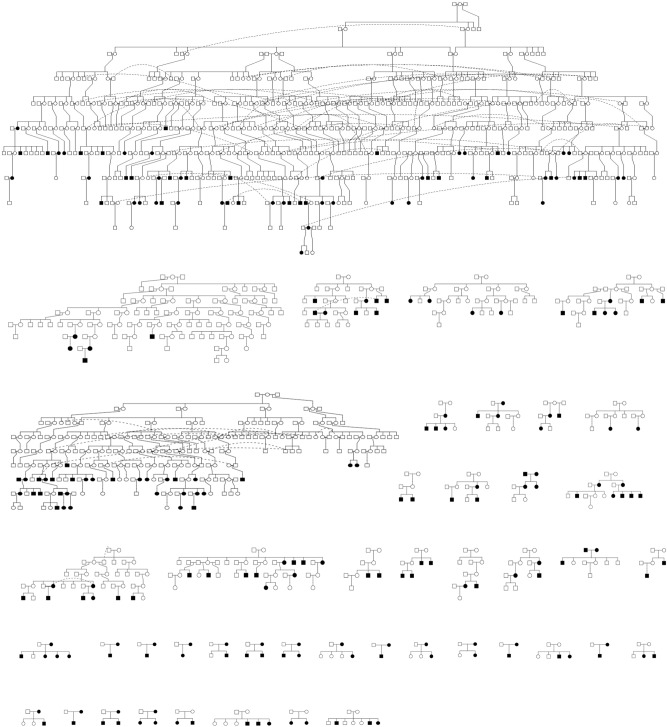
Forty-five maternal pedigrees relating to the sampled individuals examined in this study. Individual pedigrees were established for forty-five founding mothers from the Norfolk Island Core Pedigree (Fig. 1). Individuals whose mtDNA was sequenced are shown as blackened circles (females) or squares (males) and are from the lower four generations of the Norfolk Island Core Pedigree (Fig. 1). Pedigrees were generated using the Pedigree v1.4^54^, Kinship2 v1.8.5^55^ and Tidyverse v1.3.1^56^ packages in RStudio v1.4^57^.

This research used individuals from the NI Health Study and the associated NI core pedigree for research investigations at Queensland University of Technology. The NI Health Study has been well described in previous research^17–21^. The NI core pedigree contains individuals that are most closely related to the original founders. For illustration, the NI core pedigree is shown in Fig. 1. Using the NI core pedigree, maternal pedigrees were generated by establishing a list of founding mothers and tracing their maternal lines. Resultant pedigrees were generated using the Pedigree v1.4^54^, Kinship2 v1.8.5^55^ and Tidyverse v1.3.1^56^ packages. All pedigree analysis was conducted in RStudio v1.4^57^. The code utilised for the generation of pedigrees shown in Figs. 1 and 2 is available in the GitHub repository http://sirselim.github.io/presentations/mt_tracing.html. In total, 45 resultant pedigrees (families) were chosen (Fig. 2). From these families, 225 individuals (including 125 females and 100 males) were chosen for sequencing, corresponding to 345 mtDNA transmissions. All individuals chosen were from the last four generations of the NI core pedigree and relate back to the original founders.

### Whole mtGenome sequencing

The entire human mtGenome was amplified using long-range PCR with two overlapping primer sets (fragment 1: mt.569–9819, F: 5′-AACCAAACCCCAAAGACACC-3′ and R: 5′-GCCAATAATGACGTGAAGTCC-3′; and fragment 2: mt.9611–626, F: 5′-TCCCACTCCTAAACACATCC-3′ and R: 5′-TTTATGGGGTGATGTGAGCC-3′). Library preparation and sequencing was performed using an Ion Torrent high throughout sequencing protocol established in-house^58^.

### Data analysis

Data analysis was performed using the bioinformatics pipeline outlined in Harvey et al.^58^. Sequences were obtained for the entire mtGenome and were aligned in relation to the revised Cambridge Reference Sequence (rCRS)^59^ using an online tool Mitomaster to annotate variants and call haplotype information for each sample^60^.

Length variants that are known hotspots for insertion and deletions (indels) were ignored, including positions 309, 455, 463, 573, 960, 5899, 8276, 8285 and 16193 relative to the rCRS^61^. Variants in positions 16180–16183 were also ignored as reliable sequence data could not be obtained (see “Results” and “Discussion” sections).

In cases of lengthy heteroplasmy, a single dominant variant was identified. In cases of point heteroplasmy, the position was deemed heteroplasmic if the following requirements were achieved:

1. For NGS data, the minor allele frequency was greater than 20% (of total coverage). This threshold was chosen to reliably differentiate between signal and noise and reduce false positives.
2. When sequenced using Sanger sequencing, the minor allele was readily visible (a distinct peak of normal morphology was evident and white space beneath the peak could be observed without changing the chromatogram view to examine the signal closer to the baseline)^48^.
3. The minor allele was evident in two different high-quality Sanger sequences (for example, when using both forward and reverse primers).

All mutations were confirmed using Sanger sequencing. Amplification was performed in a 25 μL total reaction volume using 1.6 μL magnesium chloride, 5 μL 5X GoTaq Colourless Flexi Reaction Buffer, 0.5 μL of 10 mM dNTPs, 9.4 μL deionised water, 2 μL GoTaq Hot Start Polymerase, 2 μL of each 10 μM amplification primer and 50 ng DNA extract. Thermal cycling conditions were 95 °C for 30 s; 30 cycles of 95 °C for 30 s, 57 °C for 40 s, 72 °C for 30 s; and a 68 °C hold for 3 min. Purification of amplified products prior to sequencing was performed using ExoSAP-IT PCR Product Clean-up Reagent, using 5 μL PCR product, 4 μL deionised water, and 1 μL ExoSAP-IT. Thermal cycling conditions were as per the manufacturer’s instructions. Sequencing was performed via the BigDye Terminator v3.1 Cycle Sequencing Kit. Reactions consisted of 9.64 μL deionized water; 4 μL BigDye Terminator v3.1 Sequencing Buffer; 0.5 μL BigDye Terminator v3.1 Ready Reaction Mix; 3.2 μL sequencing primer at 10 μM; and 2.66 μL PCR product for a total reaction volume of 20 μL. Thermal cycling conditions used were: 96 °C hold for 1 min; 30 cycles of 96 °C for 10 s, 50 °C for 5 s, and 60 °C for 4 min; followed by 1 cycle of 4 °C for 5 min and 10 °C for 5 min. Sequence product purification was performed via ethanol precipitation. Sequence detection was performed by capillary electrophoresis on a 3500 Genetic Analyser using a 50 cm array, the FastSeq instrument protocol with default instrument settings. Amplification and sequencing primers are outlined in Supplementary Table S1.

The mutation rate was derived from the number of detected mutations per number of ‘meioses’ or transmission events, which is the number of cumulative generations tracing back to the maternal ancestor. Genealogical records for a sample of 222 individuals from Norfolk Island suggest that the average maternal intergenerational time is 26.9 years. The mutation rate is expressed as mutations per base pair (site) per million years (mutations/site/Myr), where the generational time was assumed to be 26.9 years. Different values of the mutation rate were estimated according to different assumptions: (1) all mutations that were detected (including heteroplasmy) were considered for the mutation rate calculation, and (2) all mutations that were detected (excluding heteroplasmy) were considered for the mutation rate calculation.

Confidence intervals (CIs: 95%) were calculated using Epitools, an online tool provided by AusVet Animal Health Services^62^. The program outputs intervals using five alternative calculation methods as described in Brown et al.^63^. The Wilson and Clopper Pearson methods were reported.

## Supplementary Information

Supplementary Information.

## Data Availability

The data that support the findings of this study have restricted access due to ethical requirements and agreements with the Norfolk Island community, and so are not publicly available. Aggregate data may, however, be made available from the authors upon request. The Norfolk genetics steering committee will assess data access requests via our GRC computational genetics group (interested researchers should contact grccomputationalgenomics@gmail.com).

## Code availability

The code utilised for this study is available in the GitHub repository https://github.com/GRC-CompGen/mitochondrial_seq_pipeline and http://sirselim.github.io/presentations/mt_tracing.html.
