# Supplementary material for: Pedigree derived mutation rate across the entire mitochondrial genome of the Norfolk Island population
Source: Sci Rep. 2022 Apr 26;12:6827. doi: 10.1038/s41598-022-10530-3 (PMC9042960; doi:10.1038/s41598-022-10530-3)
Supplement: Supplementary file 1 — Supplementary Information. [file 41598_2022_10530_MOESM1_ESM.docx]

# Supplementary Table S1: Amplification and Sequencing Primers

| Amplicon number | Amplicon size | Primer name | Primer sequence |
| --- | --- | --- | --- |
| 1 | 350 | F3 | TCACAGGTCTATCACCCTATTAACC |
|  |  | R353 | GTTTGGCAGAGATGTGTTTAAGTG |
| 2 | 535 | F362 | CAAAGAACCCTAACACCAGCCTAAC |
|  |  | R897 | GTGGCTGGCACGAAATTGACC |
| 3 | 546 | F1531 | CCCCTACGCATTTATATAGAGGAGAC |
|  |  | R2077 | GGATTTAGAGGGTTCTGTGGGC |
| 4 | 494 | F2419 | CTGTCAACCCAACACAGGCA |
|  |  | R2913 | TGGTCAAGTTATTGGATCAATTGAGT |
| 5 | 556 | F4934 | CACTCTCTCAATCTTATCCATCATAGC |
|  |  | R5490 | GGGAGATAGGTAGGAGTAGCG |
| 6 | 462 | F5828 | GTAAAAAGAGGCCTAACCCCTGTCT |
|  |  | R6290 | GTAGACTGTTCAACCTGTTCCTGCT |
| 7 | 553 | F6157 | TAATAATCGGTGCCCCCGATA |
|  |  | R6710 | TCCAAATGGTTCTTTTTTTCCGGAGTA |
| 8 | 500 | F6682 | ACTACTCCGGAAAAAAAGAACCATTT |
|  |  | R7182 | GGAAGAAAGTTAGATTTACGCCGAT |
| 9 | 495 | F7234 | CCGATGCATACACCACATGAAAC |
|  |  | R7729 | TGTTGTGAGTGTTAGGAAAAGGGCA |
| 10 | 512 | F8174 | TCTGAAATCTGTGGAGCAAACCAC |
|  |  | R8686 | AGGTTAGTTTGATTAGTCATTGTTGGG |
| 11 | 539 | F8547 | TTCATTCATTGCCCCCACAATC |
|  |  | R9086 | GGAAGGTTAATGGTTGATATTGCTAGG |
| 12 | 553 | F9228 | TATCATATAGTAAAACCCAGCCCATGA |
|  |  | R9781 | ATGCCGTCGGAAATGGTGAAG |
| 13 | 535 | F12281 | CAGCTATCCATTGGTCTTAGGC |
|  |  | R12816 | GGCGTATCATCAACTGATGAGCAAG |
| 14 | 509 | F14436 | CAGGATACTCCTCAATAGCCATCGCTG |
|  |  | R14945 | CGATTGATGAAAAGGCGGTTGAG |
| 15 | 470 | F16078 | ACAACCGCTATGTATTTCGTACATT |
|  |  | R16548 | GGGAACGTGTGGGCTATTTAGG |
